# A New Dental Journal

**Published:** 1878-01

**Authors:** 


### EDITORIAL

### A NEW DENTAL JOURNAL.

The first number of *The St. Louis Dental Quarterly*, made its appearance in October last. It is edited by Professors C. W. Spalding and H. S. Chase. This number is well gotten up, and filled with interesting matter. It is modest in its claims, it does not propose to "cover the dentists world of science and practice," but to do what it can for the furtherance of the interest of the profession, and furnish another vehicle for free discussion, it will even publish articles that are opposed to the thoughts (opinions probably) of both its editors.

Well, such a journal has long been needed, it is remarkable that nobody ever thought of this before!

Another good thing about it, is, that it is to be independent. Independent of whom? Not of the profession certainly, for it says: "We hope to have the assistance of the profession by short and interesting articles." And again it says: "Those who wish it will please remit one dollar to either of its editors."

And it is certainly not independent of the manufactures and and dealers in dental goods, and colleges, for there is nineteen pages of advertisements from about that number of different parties, bearing just about the same relation to the journal that those of other journals do to them. But then we will not be particular, and will admit that this is an independent journal.

We advise any dentists to subscribe for this journal and pay for it too. Each number will contain interesting and instruct. ive matter of far more value to the dental practitioner than the subscription for the year

